# Evaluating ChatGPT in Portal Messaging for Dementia Care: Identifying Care Partners and Exploring Acceptance

**DOI:** 10.1093/geroni/igaf122.380

**Published:** 2025-12-31

**Authors:** Kelly Gleason, Athena DeGennaro

**Affiliations:** Johns Hopkins School of Nursing, Baltimore, Maryland, United States; Johns Hopkins School of Nursing, Baltimore, Maryland, United States

## Abstract

**Introduction:**

ChatGPT, a popular large language model, is integrated in Epic’s patient portal and can create draft responses that clinicians may refine and use when answering patient messages. Application of GPT in messaging related to dementia care requires a judicious approach: persons with dementia and their care partners have distinct differences from the general population (e.g., higher care partner involvement).

**Methods:**

We conducted a two-part study. We first used a de-identified set of patient portal messages sent from persons with dementia and their care partners to assess ChatGPT’s capacity to identify the sender of the message. We used a set of 1,973 messages where the sender (patient or nonpatient) was manually coded by two independent reviewers as the gold standard. We then conducted interviews with persons with dementia and their care partners to explore their perceptions of the use of artificial intelligence in patient portal messaging. We used team-based qualitative rapid analysis encapsulate major findings across the summaries of participants’ interviews.

**Results:**

We found that ChatGPT had acceptable performance in detecting a nonpatient author of a patient portal message (0.92 area under the receiver operating curve). In qualitative interviews (n = 13), we found that care partners and persons with dementia accepted the use of artificial intelligence to draft patient portal messages provided two key conditions were met: clinician review before sending and clear transparency regarding artificial intelligence’s involvement.

**Conclusion:**

Use of ChatGPT in patient portal messaging is acceptable to care partners and persons with dementia, and may support care partner identification.

